# Using preliminary data and prospective power analyses for mid-stream revision of projected group and subgroup sizes in pragmatic patient-centered outcomes research

**DOI:** 10.1016/j.dib.2020.106529

**Published:** 2020-11-17

**Authors:** Elizabeth A. Tolley, Satya Surbhi, James E. Bailey

**Affiliations:** 1 Center for Health System Improvement, University of Tennessee Health Science Center, Memphis, TN USA; 2 Department of Preventive Medicine, University of Tennessee Health Science Center, Memphis, TN USA; 3 Division of General Internal Medicine, Department of Medicine, University of Tennessee Health Science Center, Memphis, TN USA

**Keywords:** Sample size, Statistical power, Pragmatic clinical trial, Epidemiologic research design

## Abstract

Pragmatic clinical trials are commonly used in patient-centered outcomes research to assess heterogeneity of treatment effects. Patient-Centered Outcomes Research Institute (PCORI) methodology standards for assessing heterogeneity of treatment effects are extremely rigorous, but their implementation in real-world settings can be difficult. Predicting recruitment effectiveness and subgroup characteristics is often challenging and may require mid-stream revision of projected group and subgroup sizes. Yet, little real-world data are available to demonstrate methodologically valid approaches to address situations where such revisions are necessary. These data were used for mid-stream revision of group and subgroup sizes in the Management of Diabetes in Everyday Life (MODEL) clinical trial. The planned number of randomized participants retained over the one-year study period was reduced from 800 to 581 due to recruitment difficulties among potential participants residing in rural areas. Prospective power analyses are based on the revised target of 581 participants retained and the proportions of 167 participants with various key baseline characteristics, who had been randomized in MODEL by January 2018, as reported to the Patient Center Outcomes Research Institute (PCORI) and the MODEL Data Safety and Monitoring Committee. Power calculations are based on two-sided t-tests with type-I error rates of 0.05 and the assumption that effect sizes will range from small (standardized difference = 0.36) to medium (= 0.50). The primary outcome variables are how many days in the previous week participants 1) ate healthy meals, 2) participated in at least 30 minutes of physical activity, and 3) took medications as prescribed. The POWER procedure of SAS 9.4 was used for all analyses. These data, along with the approach, can assist statisticians as they plan future pragmatic clinical trials evaluating heterogeneity of treatment effects. These data can help inform investigators, conducting patient-centered outcomes research, as they define subgroups for either confirmatory analyses for testing heterogeneity of treatment effects or for exploratory analyses where estimation of confidence bounds may be useful for generating future hypotheses. (This work was supported through a Patient-Centered Outcomes Research Institute (PCORI) Project Program Award (SC15-1503-28336), www.ClinicalTrials.gov and Identifier: NCT02957513 [1].)

## Specifications Table

**Table:** 

Subject	Epidemiology
Specific subject area	Prospective power analyses due to mid-stream revision of projected group and subgroup sizes for the purpose of estimating the projected power for detecting heterogeneity of treatment effects based on meaningful effect sizes
Type of data	Table
How data were acquired	Prospective power analyses using revised projected group and subgroup sizes and meaningful effect sizes obtained from the literature Instruments: SAS 9.4 (software)
Data format	Raw and analyzed
Parameters for data collection	Power calculations are based on two-sided t-tests with type-I error rates of 0.05 and the assumption that effect sizes will range from small (standardized difference = 0.36) to medium (= 0.50). The power analyses are based on a reduction of the number of randomized participants retained over the one-year study period from 800 to 581.
Description of data collection	The primary outcome variables are how many days in the previous week participants 1) ate healthy meals, 2) participated in at least 30 minutes of physical activity, and 3) took medications as prescribed. In the current power analyses group and subgroup sizes are based on the proportions of 167 participants with the various key baseline characteristics, who had been randomized by January 2018, as reported to PCORI and the MODEL Data Safety and Monitoring Committee. An overview of the original power analyses and sample sizes are provided at www.ClinicalTrials.gov and Identifier: NCT02957513 [1].
Data source location	University of Tennessee Health Science Center Memphis, TN 38163 United States of America 35.1408° N, 90.0306° W
Data accessibility	With the article Our expected group sizes were estimated based on a preliminary analysis of existing data in the form of attribute frequencies and proportions. The source of these proportions and frequencies were those participants enrolled and randomized in our ongoing randomized, controlled pragmatic trial, MODEL, at the time that we worked with PCORI to reduce our total sample size due to recruitment difficulties among rural residents. Currently, we are not able to provide a link to the raw data of these participants. However, based on the projected sample sizes, a reader can approximate the frequencies and proportions of attributes among the participants used for these computations. We would also add that we are obligated to make our data available upon completion of the trial, as required by PCORI. The entire data set will be available at the conclusion of the study.
Related research article	J.E. Bailey, S. Surbhi, J. Gatwood, S. Butterworth, M. Coday, S.A. Shuvo, A.A. Dashputre, I.M. Brooks, B. Binkley, C.J. Riordan, H. Steinberg, M.L. Gutierrez, L. Haley, C. Leak, E.A. Tolley, The Management of Diabetes in Everyday Life Study: Design and Methods for a Pragmatic Randomized Controlled Trial Comparing the Effectiveness of Text Messaging versus Health Coaching. Contemp Clin Trials. 2020 Sep;96:106080. doi: 10.1016/j.cct.2020.106080. Epub 2020 Jul 9. PMID: 32653539.

## Value of the Data

• Mid-stream revision of group sizes in a pragmatic randomized clinical trial should not reduce the power of tests of treatment effectiveness below accepted levels. However, any detected difference in effectiveness can be attributable to heterogeneity of treatment effects. Power analyses can reveal where tests for heterogeneity will likely have sufficient power after reduction of subgroup sizes.
• When statisticians conduct power analyses and estimate sample size requirements, they make assumptions regarding reference population characteristics and expected effect sizes. These data, along with this approach for reduction of group and subgroup sizes, can assist statisticians as they plan future studies.
• Researchers may use these projected subgroup sizes to evaluate patient attributes associated with heterogeneity of treatment effects. These data can help inform investigators as they define subgroups for either confirmatory analyses testing for heterogeneity of treatment effects or for exploratory analyses where estimation of confidence bounds may be useful for generating future hypotheses.
• Pragmatic clinical trials are commonly used in patient-centered outcomes research to assess heterogeneity of treatment effects. But predicting recruitment effectiveness and subgroup characteristics is notoriously difficult and may require mid-stream revision of projected group and subgroup sizes. These data provide an example from a real-world setting, where such revisions became necessary.
• In medically under-served areas, the negative health outcomes of patients with multiple chronic health conditions may be further exacerbated by low health literacy, high medical complexity, high social complexity, older age, limited smart phone access, and rural or suburban residency. In pragmatic clinical trials, investigators may test for heterogeneity of treatment effects affected by such patient attributes.

## 1 Data Description

Table 1 depicts power estimates for specific aim 1, which seeks to quantify the effectiveness of tailored text messaging (TM) and health coaching (HC) versus enhanced usual care (EC) in improving diabetes self-care activities related to general diet, exercise, and medication adherence. Diabetes self-care activities are operationalized by how many days in the previous week participants 1) ate healthy meals, i.e., HEALTHY EATING; 2) participated in at least 30 min of physical activity, i.e., PHYSICAL ACTIVITY; and 3) took medications as prescribed, i.e., MEDICATION ADHERENCE. Group means are the expected mean changes from baseline to the 12-month follow-up (12 month minus baseline), when 1) the EC treatment effect is no different from “usual care” or “control” and 2) the HC or TM treatment effect is the same as the projected “intervention”, based on Rosenberg et al [2] and Arora et al [3]. Group sizes are based on randomizing and retaining 581 participants. To avoid having fractions of participants allocated to the various treatments, power analyses were performed using a total sample size of 580 (232:232:116) using the POWER procedure of SAS 9.4 [4].

**Table 1:** Power Estimates for Aim 1—Quantifying the effectiveness of text-messaging (TM) and health coaching (HC) versus enhanced usual care (EC) in improving the primary outcome measures: diabetes self-care activities related to general diet, exercise, and medication adherence.

Variable	Group means (D)^a^ (12 mo – baseline)	Pooled standard deviation	Effect size (std. dev.)	Actual power b (HC or TM vs EC)
Healthy Eating	0.50, 1.60	2.2	0.50	0.992
Men				0.585
Women				0.967
Physical Activity	0.20, 1.10	2.4	0.375	0.908
Men				0.371
Women				0.812
Medication Adherence	-0.10, 0.90	2.5	0.40	0.939
Men				0.413
Women				0.859

a Group means are expected mean changes from baseline to the 12-month follow-up (12 month – baseline), when 1) the EC treatment effect is no different from “usual care” or “control”, 2) the HC or TM treatment effect is the same as the projected “intervention”, based on Rosenberg et al [2] and Arora et al [3], and 3) the final proportions of various key baseline characteristics are the same as those of 167 participants (randomized prior to January 2018).

b Group sizes are based on randomizing and retaining a total of 581 participants. Note that to avoid having fractions of participants allocated to the various treatments, power analyses were performed using a total sample size of 580 (232:232:116). Power estimates to detect differences in effectiveness by gender are based on retaining 145 men (29:58:58) and 435 women (87:174:174).

Table 2 depicts power estimates for specific aim 2, which seeks to quantify heterogeneity of treatment effects for the three primary outcome variables and test for differences in changes over 12 months (1) for the EC arm compared to either the TM or HC arm or (2) between the TM and HC arms. The 36 pre-specified contrasts of primary interest involve the direct comparison of the TM and HC treatments within the various subgroups (i.e., 2 subgroups x 6 key characteristics x 3 primary outcome variables = 36 contrasts). Diabetes self-care activities are operationalized by how many days in the previous week participants 1) ate healthy meals, i.e., HEALTHY EATING; 2) participated in at least 30 min of physical activity, i.e., PHYSICAL ACTIVITY; and 3) took medications as prescribed, i.e., MEDICATION ADHERENCE. Group means are the expected mean changes from baseline to the 12-month follow-up (12 month minus baseline), when 1) the EC treatment effect is no different from “usual care” or “control,” 2) the HC or TM treatment effect is the same as the projected “intervention”, based on Rosenberg et al [2] and Arora et al [3], and 3) the final proportions of various key baseline characteristics are the same as those of 167 participants (randomized prior to January 2018). For the HC v TM contrasts the assumption is that the effect of one of the treatment modalities (either HC or TM) in a specified subclass is not different from that of EC. Group sizes are based on randomizing and retaining 581 participants. To avoid having fractions of participants allocated to the various treatments, power analyses were performed using a total sample size of 580 (232:232:116), using the POWER procedure of SAS 9.4 [4]. Proposed contrasts with extremely low power will be used for exploratory analyses where estimation of confidence bounds may be useful for generating future hypotheses.

**Table 2:** Power Estimates for Aim 2—Testing for Heterogeneity of Treatment Effects: Contrasts between Enhanced Care (EC) and Either Text-Messaging (TM) or Health Coaching (HC) and between Text-Messaging and Health Coaching

Primary outcome and subclass variables	Group means (Δ)^a^ (12 mo – baseline)	Pooled standard deviation	Effect size (std. dev.)	Group sizes^b^ EC v TM or HC TM v HC^c^	Actual power
HealthyEating					
Health Literacy	0.5, 1.60	2.2	0.50		
Low (n=160)				32, 64	0.628
Low				64, 64	0.801
High (n=420)				84, 168	0.961
High				168, 168	0.995
Medical complexity^d^	0.5, 1.60	2.2	0.50		
Low (n=568)				114, 227	0.991
Low				227, 227	>.999
High (n=12)				2, 5	0.078
High				5, 5	0.108
Social complexity	0.5, 1.60	2.2	0.50		
Low (n=375)				75, 150	0.941
Low				150, 150	0.991
High (n=205)				41, 82	0.737
High				82, 82	0.889
Smart Phone ownership	0.5, 1.60	2.2	0.50		
Yes (n=460)				92, 184	0.974
Yes				184, 184	0.998
No (n=120)				24, 48	0.505
No				48, 48	0.679
Age <60, ≥60 years	0.5, 1.60	2.2	0.50		
Younger (n=383)				77, 153	0.949
Younger				153, 153	0.992
Older (n=197)				39, 79	0.717
Older				79, 79	0.878
Urban, Rural/Suburban	0.5, 1.60	2.2	0.50		
Urban (n=459)				92, 183	0.974
Urban				183, 183	0.998
Rural/Suburban (n=122)				24, 49	0.508
Rural/Suburban				49, 49	0.688
Physical Activity					
Health Literacy	0.20, 1.10	2.4	0.375		
Low (n=160)				32, 64	0.403
Low				64, 64	0.558
High (n=420)				84, 168	0.798
High				168, 168	0.929
Medical complexity d	0.20, 1.10	2.4	0.375		
Low (n=568)				114, 227	0.903
Low				227, 227	0.979
High (n=12)				2, 5	0.066
High				5, 5	0.082
Social complexity	0.20, 1.10	2.4	0.375		
Low (n=375)				75, 150	0.752
Low				150, 150	0.899
High (n=205)				41, 82	0.494
High				82, 82	0.665
Smart Phone ownership	0.20, 1.10	2.4	0.375		
Yes (n=460)				92, 184	0.833
Yes				184, 184	0.948
No (n=120)				24, 48	0.316
No				48, 48	0.444
Age <60, ≥60 years	0.20, 1.10	2.4	0.375		
Younger (n=383)				77, 153	0.762
Younger				153, 153	0.905
Older (n=197)				39, 79	0.476
Older				79, 79	0.649
Urban, Rural/Suburban	0.20, 1.10	2.4	0.375		
Urban (n=459)				92, 183	0.832
Urban				183, 183	0.947
Rural/Suburban (n=122)				24, 49	0.318
Rural/Suburban				49, 49	0.451
Medication Adherence					
Health Literacy	-0.10, 0.90	2.5	0.40		
Low (n=160)				32, 64	0.448
Low				64, 64	0.612
High (n=420)				84, 168	0.847
High				168, 168	0.955
Medical complexity d	-0.10, 0.90	2.5	0.40		
Low (n=568)				114, 227	0.935
Low				227, 227	0.989
High (n=12)				2, 5	0.068
High				5, 5	0.087
Social complexity	-0.10, 0.90	2.5	0.40		
Low (n=375)				75, 150	0.804
Low				150, 150	0.932
High (n=205)				41, 82	0.546
High				82, 82	0.721
Smart Phone ownership	-0.10, 0.90	2.5	0.40		
Yes (n=460)				92, 184	0.877
Yes				184, 184	0.969
No (n=120)				24, 48	0.351
No				48, 48	0.492
Age <60, ≥60 years	-0.10, 0.90	2.5	0.40		
Younger (n=383)				77, 153	0.813
Younger				153, 153	0.937
Older (n=197)				39, 79	0.527
Older				79, 79	0.705
Urban, Rural/Suburban	-0.10, 0.90	2.5	0.40		
Urban (n=459)				92, 183	0.877
Urban				183, 183	0.968
Rural/Suburban (n=122)				24, 49	0.354
Rural/Suburban				49, 49	0.500

a Group means are expected mean changes from baseline to the 12-month follow-up (12 month – baseline), when 1) the EC treatment effect is no different from “usual care” or “control”, 2) the HC or TM treatment effect is the same as the projected “intervention”, based on Rosenberg et al [2] and Arora et al [3], and 3) the final proportions of various key baseline characteristics are the same as those of 167 participants (randomized prior to January 2018).

b Group sizes are based on randomizing and retaining a total of 581 participants. Note that to avoid having fractions of participants allocated to the various treatments, power analyses were performed using a total sample size of 580 (232:232:116); subgroup sizes are based on the proportions of 167 participants (randomized prior to January 2018) and their various key baseline characteristics, as reported to PCORI and the MODEL Data Safety and Monitoring Committee.

c Of the 108 pre-specified contrasts, the most important ones are the 36 contrasts testing for differences between the adjusted one-year means of TM and HC. For the HC v TM contrasts the assumption is that the effect of one of the treatment modalities (either HC or TM) in a specified subclass is not different from that of EC. Within each key, dichotomized, baseline characteristic, these two pre-specified, confirmatory contrasts will identify and quantify the extent to which various baseline characteristics interact with the two active treatments (HC and TM), thereby producing heterogeneous treatment effects.

d Unless a sufficient number of participants with high medical complexity (i.e., high healthcare utilizers) are enrolled, randomized and retained, medical complexity as a key, baseline characteristic will be abandoned. A *post hoc* definition of high medical complexity as having two chronic conditions in addition to diabetes is under consideration.

## 2 Experimental design, materials and methods

MODEL is a pragmatic RCT designed to evaluate the comparative effectiveness of tailored text messaging (TM), health coaching (HC), and enhanced usual care (EC) interventions in a sample of African American adults with uncontrolled diabetes (DM) and multiple chronic conditions randomized to one of three treatment arms with 40% randomized to TM, 40% to HC, and 20% to enhanced usual care (EC) arm [1]. All three groups receive EC, but the EC group receives enhanced usual care alone. Baseline characteristics of participants are collected prior to randomization. Initial projected subgroup sizes were based on preliminary data, including a survey of potential participants who were then current patients at a participating clinic, with percentages as follows: low v high health literacy, 50:50; low v high medical complexity, 45:55; low v high social complexity, 55:45; smart phone v cell phone ownership, 65:35; < 60 v ≥ 60 years of age, 50:50; and rural v urban residence, 50:50. Outcomes are measured at baseline, 3 months, 6 months, and 12 months and the primary outcome is change in DM self-care activities. The primary outcome variable is operationalized by how many days in the previous week participants 1) ate healthy meals, i.e., HEALTHY EATING; 2) participated in at least 30 minutes of physical activity, i.e., PHYSICAL ACTIVITY; and 3) took medications as prescribed, i.e., MEDICATION ADHERENCE. After approximately 13 months of recruitment, the planned number of randomized participants retained over the one-year study period was reduced due to recruitment difficulties among potential participants residing in rural areas.

Sample size and power calculations are based on two-sided t-tests with type-I error rates of 0.05 and the assumption that effect sizes will range from small (standardized difference = 0.375 for PHYSICAL ACTIVITY) to medium (= 0.50 for HEALTHY EATING). The type-I error rates remain unchanged from the original power analyses, which were intended to meet PCORI Methodology Standards for Heterogeneity of Treatment Effects [5]. No adjustment is made for multiple comparisons. An alternative approach for power calculation would have been using the actual analytical model, but no data were available on which to specify a reasonable linear exponent autoregressive correlation structure for the typical repeated measures ANOVA. While the chosen approach is simplistic, it reflects published data and provides conservative power estimates. Effect sizes were based on group means and standard deviations reported from the TEAMcare trial (HC vs EC) [2] and the TExT-MED trial (TM vs EC) [3]. In order to obtain estimates of effect sizes, projected mean changes over 12-months’ follow-up from baseline (mean for 12-month follow-up minus mean for baseline) of the control and intervention arms for each primary outcome variable were obtained using results reported by Rosenberg et al [2] and Arora et al [3]. First, we averaged reported changes for the “intervention” arms from the two previous studies, when available, assuming that both the TM and HC arms of the current study would have the same average effects. Next, we assumed that the mean change from baseline in the EC arm would be similar to that observed in previous studies for the “usual care” or “control” arms. Then, for the active arms we multiplied the average mean changes at 6-months for the “intervention” arms by 2 and subtracted the mean of the “usual care” arm from projected mean change at 12-months, in order to obtain conservative estimates of the effects of the two active treatments. Thus, projected mean changes to 12 months were within ± 1 standard deviation of the respective published values for 6-month follow-up. This approach was based on the rationale that 1) most of the published mean differences reflected a study period of only 6 months; 2) in a 12-month study differences most likely would continue to increase but at a decreasing rate; and 3) the published mean differences were obtained from two separate and distinct studies on the effect of health coaching (HC) or text-messaging (TM) with no direct comparison of the two modalities of interest.

Although the methodological approach is the same as that followed for the original power analyses [1], the data reported here are based on revised power analyses conducted in January 2018, for a PCORI site visit after which the number of randomized participants retained over the one-year study period was reduced from 800 to 581. In the current power analyses group and subgroup sizes are based on the proportions of 167 participants (randomized prior to January 2018) and their various key baseline characteristics, as reported to PCORI and the MODEL Data Safety and Monitoring Committee. Power estimates were obtained using the POWER procedure of SAS 9.4 [4]. To avoid having fractions of participants allocated to the various treatments, power analyses were performed using a total sample size of 580 and projected subgroup sizes that perfectly reflected the 2:2:1 randomization scheme. The SAS code file used to compute the power of the specified hypothesis tests is contained in the supplemental materials. An overview of the original power analyses and sample sizes are provided at www.ClinicalTrials.gov (#NCT02957513) [1].

In aim 1 we propose to compare the effectiveness of each of the two active arms at one-year follow-up to that of the control arm. We do not propose to compare the overall effectiveness of the two active arms to each other, because we hypothesize that significant and meaningful heterogeneity of treatment effects and unbalanced subgroup sizes would make these overall comparisons uninterpretable. However, if no heterogeneity of treatment effects is detected, we will make those comparisons, and thereby provide a direct overall comparison of the two modalities, adjusted for unbalanced subgroup sizes. Current power estimates suggest that HEALTHY EATING is the only outcome variable for which detection of meaningful heterogeneity of treatment effects might be feasible. Thus, direct overall comparison of the two modalities will likely be made for PHYSICAL ACTIVITY and MEDICATION ADHERENCE.

For aim 1, projected mean changes for the three activities over 12-months’ follow-up were used to obtain power estimates based on estimated group sizes. Both the HC and TM arms have adequate power (all >.9) to detect meaningful changes from baseline, i.e., effectiveness, with respect to all three primary outcome variables compared to the EC arm (Table 1). Gender as a potential confounding or effect-modifier variable will need to be assessed and inclusion of gender in the model will create subgroups with unequal sizes. Initially, the expected percentage of women was > 50%, but now the female to male ratio is expected to be approximately 3:1. For males and females, we propose two pre-specified contrasts to compare EC vs TM or HC (Table 1). When gender subgroups are added to the model for Aim 1, the power of tests is expected to exceed 0.8 for women but not for men.

In aim 2 we propose to determine the contributions of six key, dichotomized, baseline characteristics (i.e., health literacy, medical complexity, social complexity, smart vs. cell phone ownership, age, and urban vs. suburban/rural residence) to the comparative effectiveness of TM, HC, and EC. For this aim we propose three pre-specified contrasts to compare HC vs TM and HC or TM vs EC. In specifying these contrasts, we followed the PCORI Methodology Standards for Heterogeneity of Treatment Effects [5]. Projected mean changes for the three activities over 12-months’ follow-up were used to obtain power estimates based on estimated subgroup sizes. Table 2 depicts power estimates for testing differences in changes over 12 months for the three primary outcome variables among participants within various key subgroups (1) for the EC arm compared to either the HC or TM arm or (2) between the HC and TM arms. Because we reduced the number of randomized participants retained over the one-year study period from 800 to 581, the power of most subgroups for Aim 2 is expected to be much less than 0.80. However, estimation of confidence bounds for interaction effects of key characteristics across treatments over time may be useful in generating hypotheses about subgroups experiencing greater or lesser effectiveness from one intervention compared to another. In addition, these results provide subgroup sizes, effect sizes, and variabilities, which can be used in designing future studies in similar populations.

## Ethics statement

The University of Tennessee Health Science Center Institutional Review Board (IRB) approved the MODEL study protocol. This pragmatic trial is registered at ClinicalTrials.gov (#NCT02957513) [1]. The participants completed screening visits in person with study staff, where the staff reviewed the consent form with them and answered questions before the participants signed the documents.

## Declaration of Competing Interest

The authors declare that they have no known competing financial interests or personal relationships which have, or could be perceived to have, influenced the work reported in this article.

## Appendix Supplementary materials

Image, application 1
