# Supplementary material for: Using preliminary data and prospective power analyses for mid-stream revision of projected group and subgroup sizes in pragmatic patient-centered outcomes research
Source: Data Brief. 2020 Nov 17;33:106529. doi: 10.1016/j.dib.2020.106529 (PMC7708786; doi:10.1016/j.dib.2020.106529)
Supplement: Supplementary file 1 [file mmc1.docx]

/***************************************************************************;

* Project: Mid-stream revision of projected group and subgroup * sizes

*

* Program name: REVISED_POWER.SAS

*

* Author: btolley

*

* Date Created: 20180129

*

* Purpose: Evaluation of power based on retention of 581

* participants reflecting a reduction from 800

* participants due to recruitment difficulties

* among rural residents

*

* Revision history:

* Date Author Revision

* 20200629 btolley Added and revised comments to improve

* clarity.

* Revised order of PROC steps to reflect

* table content.

*

*

*****************************************************************************

*/

*SCENARIO PROJECTED SAMPLE SIZES (EC:HC:TM) (116:232:232);

*Aim 1;

*BASED ON 1:2:2 RANDOMIZATION SCHEME;

* healthy eating;

**PROC** **POWER**;

twosamplemeans

groupmeans = (**0.50** **1.60**)

stddev = **2.2** **2.2**

power = **.**

GROUPNS = **116** | **232**;

**RUN**;

*physical activity;

**PROC** **POWER**;

twosamplemeans

groupmeans = (**0.20** **1.10**)

stddev = **2.4** **2.4**

power = **.**

GROUPNS = **116** | **232**;

**RUN**;

*medication adherence;

**PROC** **POWER**;

twosamplemeans

groupmeans = (-**0.10** **0.90**)

stddev = **2.5** **2.5**

power = **.**

GROUPNS = **116** | **232**;

**RUN**;

*Aim 1 Inclusion of gender;

*(EC:HC:TM) (116:232:232);

*healthy eating;

*Men: EC v HC or TM;

**PROC** **POWER**;

twosamplemeans

groupmeans = (**0.50** **1.60**)

stddev = **2.2** **2.2**

power = **.**

GROUPNS = **29** | **58**;

**RUN**;

*Women: EC v HC or TM;

**PROC** **POWER**;

twosamplemeans

groupmeans = (**0.50** **1.60**)

stddev = **2.2** **2.2**

power = **.**

GROUPNS = **87** | **174**;

**RUN**;

*physical activity;

*Men: EC v HC or TM;

**PROC** **POWER**;

twosamplemeans

groupmeans = (**0.20** **1.10**)

stddev = **2.4** **2.4**

power = **.**

GROUPNS = **29** | **58**;

**RUN**;

*Women: EC v HC or TM;

**PROC** **POWER**;

twosamplemeans

groupmeans = (**0.20** **1.10**)

stddev = **2.4** **2.4**

power = **.**

GROUPNS = **87** | **174**;

**RUN**;

*medication adherence;

*Men: EC v HC or TM;

**PROC** **POWER**;

twosamplemeans

groupmeans = (-**0.10** **0.90**)

stddev = **2.5** **2.5**

power = **.**

GROUPNS = **29** | **58**;

**RUN**;

*Women: EC v HC or TM;

**PROC** **POWER**;

twosamplemeans

groupmeans = (-**0.10** **0.90**)

stddev = **2.5** **2.5**

power = **.**

GROUPNS = **87** | **174**;

**RUN**;

*AIM 2;

*BASED ON 1:2:2 RANDOMIZATION SCHEME;

/* Subgroup sizes are based on randomizing and retaining a total of 581 participants (232:232:116)

using 580 for power analyses to avoid having decimal fractions of participants in each arm;

*/

*SCENARIO PROJECTED SAMPLE SIZES (HC:TM:EC) (232:232:116);

*HEALTHY EATING;

*EC_L V TM_L OR EC_L V HC_L & EC_H V TM_H OR EC_H V HC_H;

*TM_L V HC_L & TM_H V HC_H;

*HEALTH LITERACY LOW=160 HIGH=420;

*SUBGROUPS LOW=32, 64, 64 HIGH=84, 168, 168;

**proc** **power**;

twosamplemeans

groupmeans = (**0.50** **1.60**)

stddev = **2.2** **2.2**

power = **.**

GROUPNS = **32** | **64**;

**run**;

**proc** **power**;

twosamplemeans

groupmeans = (**0.50** **1.60**)

stddev = **2.2** **2.2**

power = **.**

GROUPNS = **64** | **64**;

**run**;

**proc** **power**;

twosamplemeans

groupmeans = (**0.50** **1.60**)

stddev = **2.2** **2.2**

power = **.**

GROUPNS = **84** | **168**;

**run**;

**proc** **power**;

twosamplemeans

groupmeans = (**0.50** **1.60**)

stddev = **2.2** **2.2**

power = **.**

GROUPNS = **168** | **168**;

**run**;

*HEALTHY EATING;

*EC_L V TM_L OR EC_L V HC_L & EC_H V TM_H OR EC_H V HC_H;

*TM_L V HC_L & TM_H V HC_H;

*medical complexity LOW=568 HIGH=12;

*SUBGROUPS LOW=114, 227, 227 HIGH=2, 5, 5;

**proc** **power**;

twosamplemeans

groupmeans = (**0.50** **1.60**)

stddev = **2.2** **2.2**

power = **.**

GROUPNS = **114** | **227**;

**run**;

**proc** **power**;

twosamplemeans

groupmeans = (**0.50** **1.60**)

stddev = **2.2** **2.2**

power = **.**

GROUPNS = **227** | **227**;

**run**;

**proc** **power**;

twosamplemeans

groupmeans = (**0.50** **1.60**)

stddev = **2.2** **2.2**

power = **.**

GROUPNS = **2** | **5**;

**run**;

**proc** **power**;

twosamplemeans

groupmeans = (**0.50** **1.60**)

stddev = **2.2** **2.2**

power = **.**

GROUPNS = **5** | **5**;

**run**;

*HEALTHY EATING;

*EC_L V TM_L OR EC_L V HC_L & EC_H V TM_H OR EC_H V HC_H;

*TM_L V HC_L & TM_H V HC_H;

*SOCIAL COMPLEXITY LOW=375 HIGH=205;

*SUBGROUPS LOW=75, 150, 150 HIGH=41, 82, 82;

**proc** **power**;

twosamplemeans

groupmeans = (**0.50** **1.60**)

stddev = **2.2** **2.2**

power = **.**

GROUPNS = **75** | **150**;

**run**;

**proc** **power**;

twosamplemeans

groupmeans = (**0.50** **1.60**)

stddev = **2.2** **2.2**

power = **.**

GROUPNS = **150** | **150**;

**run**;

**proc** **power**;

twosamplemeans

groupmeans = (**0.50** **1.60**)

stddev = **2.2** **2.2**

power = **.**

GROUPNS = **41** | **82**;

**run**;

**proc** **power**;

twosamplemeans

groupmeans = (**0.50** **1.60**)

stddev = **2.2** **2.2**

power = **.**

GROUPNS = **82** | **82**;

**run**;

*HEALTHY EATING;

*EC_L V TM_L OR EC_L V HC_L & EC_H V TM_H OR EC_H V HC_H;

*TM_L V HC_L & TM_H V HC_H;

*SMART PHONE YES=460 NO=120;

*SUBGROUPS YES=92, 184, 184 NO=24, 48, 48;

**proc** **power**;

twosamplemeans

groupmeans = (**0.50** **1.60**)

stddev = **2.2** **2.2**

power = **.**

GROUPNS =**92** | **184**;

**run**;

**proc** **power**;

twosamplemeans

groupmeans = (**0.50** **1.60**)

stddev = **2.2** **2.2**

power = **.**

GROUPNS = **184** | **184**;

**run**;

**proc** **power**;

twosamplemeans

groupmeans = (**0.50** **1.60**)

stddev = **2.2** **2.2**

power = **.**

GROUPNS = **24** | **48**;

**run**;

**proc** **power**;

twosamplemeans

groupmeans = (**0.50** **1.60**)

stddev = **2.2** **2.2**

power = **.**

GROUPNS = **48** | **48**;

**run**;

*HEALTHY EATING;

*EC_L V TM_L OR EC_L V HC_L & EC_H V TM_H OR EC_H V HC_H;

*TM_L V HC_L & TM_H V HC_H;

*AGE <60=383 >=60=197;

*SUBGROUPS LOW=77, 153, 153 HIGH=39, 79, 79;

**proc** **power**;

twosamplemeans

groupmeans = (**0.50** **1.60**)

stddev = **2.2** **2.2**

power = **.**

GROUPNS = **77** | **153**;

**run**;

**proc** **power**;

twosamplemeans

groupmeans = (**0.50** **1.60**)

stddev = **2.2** **2.2**

power = **.**

GROUPNS = **153** | **153**;

**run**;

**proc** **power**;

twosamplemeans

groupmeans = (**0.50** **1.60**)

stddev = **2.2** **2.2**

power = **.**

GROUPNS = **39** | **79**;

**run**;

**proc** **power**;

twosamplemeans

groupmeans = (**0.50** **1.60**)

stddev = **2.2** **2.2**

power = **.**

GROUPNS = **79** | **79**;

**run**;

*HEALTHY EATING;

*EC_L V TM_L OR EC_L V HC_L & EC_H V TM_H OR EC_H V HC_H;

*TM_L V HC_L & TM_H V HC_H;

*residence urban=459 suburban/rural=122;

*SUBGROUPS LOW=92, 183, 183 HIGH=24, 49, 49;

**proc** **power**;

twosamplemeans

groupmeans = (**0.50** **1.60**)

stddev = **2.2** **2.2**

power = **.**

GROUPNS = **92** | **183**;

**run**;

**proc** **power**;

twosamplemeans

groupmeans = (**0.50** **1.60**)

stddev = **2.2** **2.2**

power = **.**

GROUPNS = **183** | **183**;

**run**;

**proc** **power**;

twosamplemeans

groupmeans = (**0.50** **1.60**)

stddev = **2.2** **2.2**

power = **.**

GROUPNS = **24** | **49**;

**run**;

**proc** **power**;

twosamplemeans

groupmeans = (**0.50** **1.60**)

stddev = **2.2** **2.2**

power = **.**

GROUPNS = **49** | **49**;

**run**;

*PHYSICAL ACTIVITY;

*EC_L V TM_L OR EC_L V HC_L & EC_H V TM_H OR EC_H V HC_H;

*TM_L V HC_L & TM_H V HC_H;

*HEALTH LITERACY LOW=160 HIGH=420;

*SUBGROUPS LOW=32, 64, 64 HIGH=84, 168, 168;

**proc** **power**;

twosamplemeans

groupmeans = (**0.20** **1.10**)

stddev = **2.4** **2.4**

power = **.**

GROUPNS = **32** | **64**;

**run**;

**proc** **power**;

twosamplemeans

groupmeans = (**0.20** **1.10**)

stddev = **2.4** **2.4**

power = **.**

GROUPNS = **64** | **64**;

**run**;

**proc** **power**;

twosamplemeans

groupmeans = (**0.20** **1.10**)

stddev = **2.4** **2.4**

power = **.**

GROUPNS = **84** | **168**;

**run**;

**proc** **power**;

twosamplemeans

groupmeans = (**0.20** **1.10**)

stddev = **2.4** **2.4**

power = **.**

GROUPNS = **168** | **168**;

**run**;

*PHYSICAL ACTIVITY;

*EC_L V TM_L OR EC_L V HC_L & EC_H V TM_H OR EC_H V HC_H;

*TM_L V HC_L & TM_H V HC_H;

*medical complexity LOW=12 HIGH=568;

*SUBGROUPS LOW=114, 227, 227 HIGH=2, 5, 5;

**proc** **power**;

twosamplemeans

groupmeans = (**0.20** **1.10**)

stddev = **2.4** **2.4**

power = **.**

GROUPNS = **114** | **227**;

**run**;

**proc** **power**;

twosamplemeans

groupmeans = (**0.20** **1.10**)

stddev = **2.4** **2.4**

power = **.**

GROUPNS = **227** | **227**;

**run**;

**proc** **power**;

twosamplemeans

groupmeans = (**0.20** **1.10**)

stddev = **2.4** **2.4**

power = **.**

GROUPNS = **2** | **5**;

**run**;

**proc** **power**;

twosamplemeans

groupmeans = (**0.20** **1.10**)

stddev = **2.4** **2.4**

power = **.**

GROUPNS = **5** | **5**;

**run**;

*PHYSICAL ACTIVITY;

*EC_L V TM_L OR EC_L V HC_L & EC_H V TM_H OR EC_H V HC_H;

*TM_L V HC_L & TM_H V HC_H;

*SOCIAL COMPLEXITY LOW=375 HIGH=205;

*SUBGROUPS LOW=75, 150, 150 HIGH=41, 82, 83;

**proc** **power**;

twosamplemeans

groupmeans = (**0.20** **1.10**)

stddev = **2.4** **2.4**

power = **.**

GROUPNS = **75** | **150**;

**run**;

**proc** **power**;

twosamplemeans

groupmeans = (**0.20** **1.10**)

stddev = **2.4** **2.4**

power = **.**

GROUPNS = **150** | **150**;

**run**;

**proc** **power**;

twosamplemeans

groupmeans = (**0.20** **1.10**)

stddev = **2.4** **2.4**

power = **.**

GROUPNS = **41** | **82**;

**run**;

**proc** **power**;

twosamplemeans

groupmeans = (**0.20** **1.10**)

stddev = **2.4** **2.4**

power = **.**

GROUPNS = **82** | **82**;

**run**;

*PHYSICAL ACTIVITY;

*EC_L V TM_L OR EC_L V HC_L & EC_H V TM_H OR EC_H V HC_H;

*TM_L V HC_L & TM_H V HC_H;

*SMART PHONE YES=460 NO=120;

*SUBGROUPS Y=92, 184, 184 N=24, 48, 48;

**proc** **power**;

twosamplemeans

groupmeans = (**0.20** **1.10**)

stddev = **2.4** **2.4**

power = **.**

GROUPNS = **92** | **184**;

**run**;

**proc** **power**;

twosamplemeans

groupmeans = (**0.20** **1.10**)

stddev = **2.4** **2.4**

power = **.**

GROUPNS = **184** | **184**;

**run**;

**proc** **power**;

twosamplemeans

groupmeans = (**0.20** **1.10**)

stddev = **2.4** **2.4**

power = **.**

GROUPNS = **24** | **48**;

**run**;

**proc** **power**;

twosamplemeans

groupmeans = (**0.20** **1.10**)

stddev = **2.4** **2.4**

power = **.**

GROUPNS = **48** | **48**;

**run**;

*PHYSICAL ACTIVITY;

*EC_L V TM_L OR EC_L V HC_L & EC_H V TM_H OR EC_H V HC_H;

*TM_L V HC_L & TM_H V HC_H;

*AGE <60=383 >=60=197;

*SUBGROUPS <65=77, 153, 153 >=65=39, 79, 79;

**proc** **power**;

twosamplemeans

groupmeans = (**0.20** **1.10**)

stddev = **2.4** **2.4**

power = **.**

GROUPNS = **77** | **153**;

**run**;

**proc** **power**;

twosamplemeans

groupmeans = (**0.20** **1.10**)

stddev = **2.4** **2.4**

power = **.**

GROUPNS = **153** | **153**;

**run**;

**proc** **power**;

twosamplemeans

groupmeans = (**0.20** **1.10**)

stddev = **2.4** **2.4**

power = **.**

GROUPNS = **39** | **79**;

**run**;

**proc** **power**;

twosamplemeans

groupmeans = (**0.20** **1.10**)

stddev = **2.4** **2.4**

power = **.**

GROUPNS = **79** | **79**;

**run**;

*PHYSICAL ACTIVITY;

*EC_L V TM_L OR EC_L V HC_L & EC_H V TM_H OR EC_H V HC_H;

*TM_L V HC_L & TM_H V HC_H;

*residence urban=459 suburban/rural=122;

*SUBGROUPS urban=92, 183, 183 suburban/rural=24, 49, 49;

**proc** **power**;

twosamplemeans

groupmeans = (**0.20** **1.10**)

stddev = **2.4** **2.4**

power = **.**

GROUPNS = **92** | **183**;

**run**;

**proc** **power**;

twosamplemeans

groupmeans = (**0.20** **1.10**)

stddev = **2.4** **2.4**

power = **.**

GROUPNS = **183** | **183**;

**run**;

**proc** **power**;

twosamplemeans

groupmeans = (**0.20** **1.10**)

stddev = **2.4** **2.4**

power = **.**

GROUPNS = **24** | **49**;

**run**;

**proc** **power**;

twosamplemeans

groupmeans = (**0.20** **1.10**)

stddev = **2.4** **2.4**

power = **.**

GROUPNS = **49** | **49**;

**run**;

*MEDICATION ADHERENCE;

*EC_L V TM_L OR EC_L V HC_L & EC_H V TM_H OR EC_H V HC_H;

*TM_L V HC_L & TM_H V HC_H;

*HEALTH LITERACY LOW=140 HIGH=320;

*SUBGROUPS LOW=32, 64, 64 HIGH=84, 168, 168;

**proc** **power**;

twosamplemeans

groupmeans = (-**0.10** **0.90**)

stddev = **2.5** **2.5**

power = **.**

GROUPNS = **32** | **64**;

**run**;

**proc** **power**;

twosamplemeans

groupmeans = (-**0.10** **0.90**)

stddev = **2.5** **2.5**

power = **.**

GROUPNS = **64** | **64**;

**run**;

**proc** **power**;

twosamplemeans

groupmeans = (-**0.10** **0.90**)

stddev = **2.5** **2.5**

power = **.**

GROUPNS = **84** | **168**;

**run**;

**proc** **power**;

twosamplemeans

groupmeans = (-**0.10** **0.90**)

stddev = **2.5** **2.5**

power = **.**

GROUPNS = **168** | **168**;

**run**;

*MEDICATION ADHERENCE;

*EC_L V TM_L OR EC_L V HC_L & EC_H V TM_H OR EC_H V HC_H;

*TM_L V HC_L & TM_H V HC_H;

*medical complexity LOW= H=;

*SUBGROUPS LOW=114, 227, 227 HIGH=2, 5, 5;

**proc** **power**;

twosamplemeans

groupmeans = (-**0.10** **0.90**)

stddev = **2.5** **2.5**

power = **.**

GROUPNS = **114** | **227**;

**run**;

**proc** **power**;

twosamplemeans

groupmeans = (-**0.10** **0.90**)

stddev = **2.5** **2.5**

power = **.**

GROUPNS = **227** | **227** ;

**run**;

**proc** **power**;

twosamplemeans

groupmeans = (-**0.10** **0.90**)

stddev = **2.5** **2.5**

power = **.**

GROUPNS = **2** | **5** ;

**run**;

**proc** **power**;

twosamplemeans

groupmeans = (-**0.10** **0.90**)

stddev = **2.5** **2.5**

power = **.**

GROUPNS = **5** | **5** ;

**run**;

*MEDICATION ADHERENCE;

*EC_L V TM_L OR EC_L V HC_L & EC_H V TM_H OR EC_H V HC_H;

*TM_L V HC_L & TM_H V HC_H;

*SOCIAL COMPLEXITY LOW=375 H=205;

*SUBGROUPS LOW=75, 150, 150 HIGH=41, 82, 82;

**proc** **power**;

twosamplemeans

groupmeans = (-**0.10** **0.90**)

stddev = **2.5** **2.5**

power = **.**

GROUPNS = **75** | **150**;

**run**;

**proc** **power**;

twosamplemeans

groupmeans = (-**0.10** **0.90**)

stddev = **2.5** **2.5**

power = **.**

GROUPNS = **150** | **150**;

**run**;

**proc** **power**;

twosamplemeans

groupmeans = (-**0.10** **0.90**)

stddev = **2.5** **2.5**

power = **.**

GROUPNS = **41** | **82**;

**run**;

**proc** **power**;

twosamplemeans

groupmeans = (-**0.10** **0.90**)

stddev = **2.5** **2.5**

power = **.**

GROUPNS = **82** | **82**;

**run**;

*MEDICATION ADHERENCE;

*EC_L V TM_L OR EC_L V HC_L & EC_H V TM_H OR EC_H V HC_H;

*TM_L V HC_L & TM_H V HC_H;

*SMART PHONE YES=460 NO=120;

*SUBGROUPS YES=92, 184, 184 NO=24, 48, 48;

**proc** **power**;

twosamplemeans

groupmeans = (-**0.10** **0.90**)

stddev = **2.5** **2.5**

power = **.**

GROUPNS = **92** | **184**;

**run**;

**proc** **power**;

twosamplemeans

groupmeans = (-**0.10** **0.90**)

stddev = **2.5** **2.5**

power = **.**

GROUPNS = **184** | **184**;

**run**;

**proc** **power**;

twosamplemeans

groupmeans = (-**0.10** **0.90**)

stddev = **2.5** **2.5**

power = **.**

GROUPNS = **24** | **48**;

**run**;

**proc** **power**;

twosamplemeans

groupmeans = (-**0.10** **0.90**)

stddev = **2.5** **2.5**

power = **.**

GROUPNS = **48** | **48**;

**run**;

*MEDICATION ADHERENCE;

*EC_L V TM_L OR EC_L V HC_L & EC_H V TM_H OR EC_H V HC_H;

*TM_L V HC_L & TM_H V HC_H;

*AGE <60=383 >=60=197;

*SUBGROUPS <60=77, 153, 153 >=60=39, 79, 79;

**proc** **power**;

twosamplemeans

groupmeans = (-**0.10** **0.90**)

stddev = **2.5** **2.5**

power = **.**

GROUPNS = **77** | **153**;

**run**;

**proc** **power**;

twosamplemeans

groupmeans = (-**0.10** **0.90**)

stddev = **2.5** **2.5**

power = **.**

GROUPNS = **153** | **153**;

**run**;

**proc** **power**;

twosamplemeans

groupmeans = (-**0.10** **0.90**)

stddev = **2.5** **2.5**

power = **.**

GROUPNS = **39** | **79**;

**run**;

**proc** **power**;

twosamplemeans

groupmeans = (-**0.10** **0.90**)

stddev = **2.5** **2.5**

power = **.**

GROUPNS = **79** | **79**;

**run**;

*MEDICATION ADHERENCE;

*EC_L V TM_L OR EC_L V HC_L & EC_H V TM_H OR EC_H V HC_H;

*TM_L V HC_L & TM_H V HC_H;

*residence urban=459 suburban/rural=122;

*SUBGROUPS urban=92, 183, 183 suburban/rural=24, 49, 49;

**proc** **power**;

twosamplemeans

groupmeans = (-**0.10** **0.90**)

stddev = **2.5** **2.5**

power = **.**

GROUPNS = **92** | **183**;

**run**;

**proc** **power**;

twosamplemeans

groupmeans = (-**0.10** **0.90**)

stddev = **2.5** **2.5**

power = **.**

GROUPNS = **183** | **183**;

**run**;

**proc** **power**;

twosamplemeans

groupmeans = (-**0.10** **0.90**)

stddev = **2.5** **2.5**

power = **.**

GROUPNS = **24** | **49**;

**run**;

**proc** **power**;

twosamplemeans

groupmeans = (-**0.10** **0.90**)

stddev = **2.5** **2.5**

power = **.**

GROUPNS = **49** | **49**;

**run**;
